# South African mothers’ immediate and 5-year retrospective reports of drinking alcohol during pregnancy

**DOI:** 10.1371/journal.pone.0231518

**Published:** 2020-04-16

**Authors:** Kodi B. Arfer, Mary J. O’Connor, Mark Tomlinson, Mary Jane Rotheram-Borus

**Affiliations:** 1 Icahn School of Medicine, Mount Sinai Health System, New York, New York, United States of America; 2 Semel Institute, University of California Los Angeles, Los Angeles, California, United States of America; 3 Department of Psychology, Stellenbosch University, Stellenbosch, South Africa; 4 Global Center for Children and Families, University of California Los Angeles, Los Angeles, California, United States of America; Medical College of Wisconsin, UNITED STATES

## Abstract

Prenatal alcohol-drinking is often measured with self-report, but it is unclear whether mothers give more accurate answers when asked while pregnant or some time after their pregnancy. There is also the question of whether to measure drinking in a dichotomous or continuous fashion. We sought to examine how the timing and scale of self-reports affected the content of reports. From a sample of 576 black mothers around Cape Town, South Africa, we compared prenatal reports of prenatal drinking with 5-year retrospective reports, and dichotomous metrics (drinking or abstinent) with continuous metrics (fluid ounces of absolute alcohol drunk per day). Amounts increased over the 5-year period, whereas dichotomous measures found mothers less likely to report drinking later. All four measures were weakly associated with birth weight, birth height, child head circumference soon after birth, and child intelligence at age 5. Furthermore, neither reporting time nor the scale of measurement were consistently related to the strengths of these associations. Our results point to problems with self-report, particularly with this population, but we recommend post-birth continuous measures as the best of the group for their flexibility and their consistency with previous research.

## Introduction

Drinking during pregnancy can lead to fetal alcohol spectrum disorder (FASD), a spectrum of conditions that can lead to a wide variety of detrimental consequences for the health of a child [1]. Measuring how much expectant mothers drink is necessary to study, monitor, and prevent FASD, but mothers may not report their drinking to investigators [2]. Of 180 countries, FASD is most common in South Africa, with 5.8% of people affected [3].

Are mothers’ self-reports of prenatal drinking most accurate during, shortly after, or long after the pregnancy in question? Studies from Cleveland and Detroit hospitals asking mothers about prenatal drinking both during pregnancy and from 1 to 14 years after birth have found that mothers’ reports of how much they drink are much more likely to increase than decrease [4, 5, 6]. In other words, within subjects, mothers report greater alcohol consumption when interviewed retrospectively than when interviewed contemporaneously. However, past results are less consistent when drinking data is dichotomized into any drinking versus no drinking, rather than represented as ounces of alcohol. Comparing the abstinent consistency rate (the proportion of mothers who said they abstained retrospectively, among those who had also said they abstained contemporaneously) to the drinking consistency rate, one study [6] found greater abstinent consistency (80% vs. 70%), another [4] found greater drinking consistency (61% vs. 78%), and another [5] found very similar rates (78% vs. 80%). It is thus unclear whether retrospective report makes mothers more or less likely to report drinking during pregnancy. Furthermore, it is unclear how well these results generalize outside the United States. In South Africa, prenatal drinking is common [7], as attested by the high rate of FASD mentioned above, and mothers may regard prenatal drinking as commonplace and socially acceptable [8, 9]. On the other hand, Cape Town residents tend to see alcoholism as quite dangerous and indicative of weak moral character [10].

Why would one use a dichotomous measure of prenatal drinking rather than a continuous one? A dichotomous measure is easy to administer: it requires no prop beverage containers, definition of a standard drink, distinction between types of beverages because of their different concentrations of alcohol, or discernment of mothers’ drinking patterns. Besides reducing potential variability in how mothers interpret questions, a dichotomous measure is less dependent on memory. A priori, people seem likely to misremember exactly how much, when, or what they drank, but should have little trouble remembering whether they drank at all. Finally, while there may be dose-dependent effects of alcohol on fetuses, the nature of any such relationships is unclear [11], and the consensus of researchers is that no amount of alcohol during pregnancy is safe [1, contrast 11], so preventing prenatal drinking entirely, rather than merely reducing frequencies or amounts to a known nonzero safe level, is typically the goal of interventions.

In this study, we compare prenatal reports of prenatal drinking with 5-year retrospective reports among mothers in the Western Cape. We also compare dichotomous to continuous measures of drinking. Finally, we compare how these various ways of measuring drinking (during or after pregnancy, and dichotomously or continuously) are associated with child health. The overall goal is to better understand the strengths and weaknesses of these strategies, for the benefit of future investigation into prenatal drinking. By clarifying whether self-reports of drinking are more useful when collected during or after pregnancy, or coded dichotomously or continuously, we aimed to help future investigators decide which measures to collect and focus on in their analyses. Our study is the first to provide a head-to-head comparison of all four methods.

## Method

We use data from a cluster-randomized controlled trial of a mentor intervention for new mothers and their babies in the area of Cape Town, South Africa (see [12], ClinicalTrials.gov registration number NCT00972699). The study was approved by the University of California Los Angeles South General Institutional Review Board, protocol identifier IRB#10–000386, and by the Research Ethics Committee of Stellenbosch University of South Africa. All mothers provided written informed consent. The data for this study is provided as S1 File, and both the data and the analysis code are available at http://arfer.net/projects/philani.

Mothers were recruited for the study from 26 neighborhoods (see 12 for details on this and other aspects of the design and conduct of the overarching clinical trial). Neighborhoods were grouped into pairs on the basis of population; duration of household residence; number of child-care center, shops, and schools; and nonproximity. Within pairs, neighborhoods were randomly assigned one of the two conditions (experimental and control), because outcomes could not be expected to be entirely independent of whether a mother next door received the experimental treatment (home visits). Mothers were recruited from May 2009 to September 2010. Pregnant black women aged 18 or more living in target neighborhoods and able to give informed consent were asked to enroll in the study, and 98% agreed. Trained interviewers, who were other township women, recorded data with mobile phones. The data was sent to servers managed by Cape Town-based Mobenzi Technologies (RF) (Pty) Ltd, and managed and processed with SAS (SAS Institute, Inc.) and Hy (http://hylang.org).

Mothers in both conditions received healthcare at clinics, including HIV testing, antenatal visits, and maternal health assessments. Mothers in the experimental condition additionally received regular home visits from another mother who was a trained community health worker and a positive deviant (i.e., a person who has had better outcomes than most of her peers), having had a healthy baby herself. In this paper, we consider only the mothers in the control group; including the experimental group would lead to multiple publication of the same results.

Our analysis draws primarily from assessments with AUDIT-C-inspired [13] interview questions at two timepoints, baseline and 5 years after birth; we chose an interval of 5 years because of an earlier finding of significant report–outcome associations at 5 years [14]. Interviewers made multiple attempts to recontact each mother at every follow-up timepoint. At the time of the baseline assessment (T1), mothers were 3 to 40 weeks pregnant (mean 26 weeks). Mothers were asked "How often did you use alcohol in the month before you found out you were pregnant?" and "During the month before you found out you were pregnant, counting all types of alcohol combined, how many drinks did you USUALLY have on days when you drank alcohol?". (The second question was skipped if the answer to the first was "Never". We asked mothers about the month before they recognized their pregnancy because we expected they would be more candid about this period, that responses would be more strongly related to outcomes, [15], and that they would notice their pregnancies relatively late, [16], indeed, at T1, mothers reported they had been a median of 7 weeks pregnant by the time they noticed they were pregnant. We also asked mothers about drinking after they had recognized their pregnancy, but those questions are not analyzed here.) At the 5-year follow-up (T2), mothers were asked the same questions, except the word "pregnant" was replaced with "pregnant with" followed by the child’s name. To define for the mother how large a drink was, the interviewer showed prop beverage containers including a beer bottle, a 250-mL wine glass, and a shot glass; we aimed to represent the American standard drink of 0.6 fl oz (14 g) of ethanol. Table 1 shows the response options for the two questions and how they are coded. For a dichotomous measure of drinking, we code mothers as abstinent if they answered "Never" and drinking otherwise. To compute fluid ounces of absolute alcohol drunk per day (AA/day), a measure we chose to aid comparison with previous research, we multiply 0.6 by the numeric frequency code (from [17] by the numeric amount code. Note that all responses and codes refer to the time periods about which mothers were asked; we do not assess mothers’ drinking on the day they were interviewed.

**Table 1:** The response options for the two drinking questions, and how we represent them as numeric codes. Frequency codes are based on 17. Amount codes are the means of the listed numbers of drinks for each option.

Response option	Code
Drinking frequency	
Never	0.00
Less than once a month	0.02
Once a month	0.05
2 to 3 times a month	0.10
Once a week	0.20
2 times a week	0.50
3 to 4 times a week	0.60
Nearly every day	0.70
Every day	1.00
Number of drinks	
1 or 2	1.50
3 or 4	3.50
5 or 6	5.50
7,8 or 9	8.00
10 or more	10.00

Mothers were also asked questions about drinking during pregnancy at another timepoint T1.5, between T1 and T2, after birth and before the children were more than 4 months old. These questions, however, asked about "the last month, before your baby was born". Because they are not directly comparable to the T1 and T2 questions analyzed here, we exclude them from analysis.

We consider four outcomes. At T1.5, mothers reported the weight and height of their children at birth. We measured the current circumference of children’s heads, and calculated their sex- and age-specific *z*-scores using WHO norms. Low weight, height, and head circumference are common diagnostic criteria for FASD [18]. At T2, children were administered the Kaufman Assessment Battery for Children [19]. Our analyses use the Mental Processing Index (MPI), which measures general mental processing ability (i.e., intelligence) and excludes an assessment of acquired knowledge. Like traditional intelligence measures, the MPI is normed to have a mean of 100 and an SD of 15. Intellectual impairment is given as a diagnostic criterion for FASD by [20].

## Results

Table 2 compares reports of drinking at T1 and T2. We use the Jeffreys interval for proportions for its short length and good coverage [21], and we use bootstrapping to compute a confidence interval for the mean difference in amounts so that we need not assume a specific distribution. (Specifically, we use the bias-corrected and accelerated bootstrap of [22], which is similar in effect to the percentile bootstrap but incorporates corrections for any bias or skewness in the bootstrap distribution.) Coding drinking dichotomously, we see that mothers reported drinking at a slightly higher rate at T1 than T2, and mothers who stated they were abstinent at T1 were much more likely to repeat their answer at T2 than mothers who stated they drank at T1. By contrast, when we code drinking in terms of AA/day, means are higher at T2 than T1, and mothers were slightly more likely to increase their reported amount of drinking than decrease it; the mean change was positive, at .12 AA/day. The doubling of between-subjects means (.10 to .20 AA/day) is mirrored by a fourfold increase in the proportion of mothers who report drinking at least 3 drinks/day (1.8 AA/day), from 1.0% (5 mothers) to 4.1% (18 mothers).

**Table 2:** Mothers’ reports of drinking at the two timepoints, baseline (T1) and 5 years after the child’s birth (T2). Mothers who are "consistent" are those who gave the same dichotomous answers at the two timepoints. AA/day = fluid ounces of absolute alcohol per day, CI = confidence interval.

Dichotomous (drinking vs. abstinent)	
Mothers reporting, either timepoint	576
Percent drinking, T1	26%
Percent drinking, T2	21%
Mothers reporting, both timepoints	366
Percent consistent, among abstinent at T1	92%
95% Jeffreys CI	[88%, 95%]
Percent consistent, among drinking at T1	63%
95% Jeffreys CI	[53%, 72%]
Continuous (AA/day)	
Mothers reporting, either timepoint	576
Mean, T1	0.10
Mean, T2	0.20
Maximum, T1	3.60
Maximum, T2	3.60
Mothers reporting, both timepoints	365
Percent increasing from T1 to T2	18%
Percent decreasing from T1 to T2	12%
Mean change	0.12
95% bootstrap CI	[0.07, 0.19]
Mean absolute change	0.20

To investigate sources of inconsistency, we examine regression models with T2 drinking as the dependent variable and the following predictors: T1 drinking, pregnancy after the study birth (yes or no), and neighborhood. We omit the intercept, instead giving each of the 12 neighborhoods its own dummy variable. We fit one model for dichotomous drinking, using logistic regression (with drinking as the positive outcome), and one model for continuous drinking, using Tobit regression to account for zero inflation (the Tobit model treats the dependent variable as left-censored at 0; i.e., it treats the observed dependent variable as arising from an unbounded latent variable that is observed as 0 when it falls below 0).

The model coefficients are shown in Table 3. Both models show a strong association of T1 drinking reports with T2 drinking reports. In the continuous model, the coefficient of T1 drinking is 1.92, indicating that mothers tend to report drinking almost twice as much at T2 as they did at T1. A new pregnancy is slightly positively associated with drinking at T2. Neighborhood effects range from neighborhood 05b, which is associated with the least drinking in both models, to neighborhood 15b, which is associated with the most. Perhaps uncoincidentally, neighborhood 05b has the largest sample and 15b the smallest.

**Table 3:** Coefficients of regression models of T2 drinking, with 95% confidence intervals. The discrete model uses logistic regression, so coefficients are on the scale of log odds. The continuous model uses Tobit regression, so coefficients are on the same scale as ordinary linear regression. The rightmost column shows per-neighborhood sample sizes.

Predictor	Discrete	Continuous	n
(Sample size)	366	366	
T1 drinking	+2.99 [+2.37, +3.66]	+1.92 [+1.26, +2.58]	
Pregnant since birth	+0.16 [-0.48, +0.81]	+0.15 [-0.32, +0.63]	
Neighborhood 01a	-3.27 [-4.71, -2.05]	-2.01 [-3.03, -0.99]	30
Neighborhood 01b	-1.74 [-2.75, -0.84]	-1.35 [-2.10, -0.60]	37
Neighborhood 02b	-2.15 [-3.21, -1.21]	-1.47 [-2.29, -0.65]	36
Neighborhood 04b	-2.92 [-4.37, -1.74]	-1.88 [-2.85, -0.91]	30
Neighborhood 05b	-3.28 [-4.68, -2.12]	-2.09 [-3.04, -1.13]	40
Neighborhood 07a	-2.40 [-3.52, -1.40]	-1.71 [-2.55, -0.87]	33
Neighborhood 08a	-3.14 [-4.65, -1.81]	-1.58 [-2.63, -0.54]	21
Neighborhood 10a	-2.77 [-4.20, -1.61]	-1.99 [-2.96, -1.02]	31
Neighborhood 11b	-2.61 [-3.98, -1.40]	-1.31 [-2.26, -0.36]	24
Neighborhood 14b	-2.15 [-3.24, -1.16]	-1.10 [-1.88, -0.32]	31
Neighborhood 15b	-1.36 [-2.77, -0.07]	-0.40 [-1.35, +0.55]	15
Neighborhood 16a	-2.62 [-3.73, -1.64]	-1.53 [-2.32, -0.74]	38

We next use ordinary linear regression models to examine how prenatal versus 5-year reports and dichotomous versus continuous coding affect the association of prenatal drinking with child health. We consider four different outcomes: weight at birth (kg), height at birth (cm), head circumference after birth (*z*-score from age- and sex-specific norms), and intelligence (on the MPI scale, which is normed to have mean 100 and SD 15). The predictor variables are drinking, pregnancy after the study birth (yes or no), neighborhood, and child sex. (Sex is omitted for the head-circumference models, since these scores are already sex-normed.) Outcomes are unstandardized, but continuous drinking scores are standardized to SD 1/2 to put their coefficients on the same scale as that of the discrete drinking scores [23]. Finally, for birth height, the highest value (78 cm) seems to be an outlier, so we replace it with the second-highest value (61 cm).

Table 4 shows the coefficient of the drinking predictor for each of these 16 models. (See the S1 Table for the coefficients of the other predictors, as well as *R*2 values.) Most coefficients are negative, as expected. Their sizes, however, are generally small. Regardless of timepoint and scale of drinking measurement, the absolute coefficient of drinking does not exceed 80 g for birth weight, 5 mm for birth height, 0.4 *z*-units for head circumference, or 1.5 MPI points for intelligence.

**Table 4:** Drinking coefficients of regression models for outcomes, with 95% confidence intervals, using prenatal drinking reports from baseline (T1) or 5 years after the child’s birth (T2), and measuring drinking on a dichotomous (drinking or not drinking) or continuous (fluid ounces of absolute alcohol per day) scale. The outcomes considered are birth weight, birth height, head circumference after birth (WHO *z*-score for age and sex) and year-5 child intelligence (Kaufman mental processing index). *n* is the effective sample size, the number of cases with no missing values.

Outcome	Time	Scale	*n*	Effect of drinking
Weight (kg)	T1	Discrete	339	-0.07 [-0.21, +0.07]
Weight (kg)	T1	Continuous	338	-0.05 [-0.17, +0.07]
Weight (kg)	T2	Discrete	354	-0.08 [-0.23, +0.08]
Weight (kg)	T2	Continuous	354	-0.08 [-0.20, +0.05]
Height (cm)	T1	Discrete	215	-0.41 [-1.61, +0.79]
Height (cm)	T1	Continuous	214	-0.13 [-1.14, +0.88]
Height (cm)	T2	Discrete	230	-0.07 [-1.41, +1.26]
Height (cm)	T2	Continuous	230	+0.08 [-0.93, +1.08]
Head circumference (*z*-score)	T1	Discrete	365	-0.26 [-0.71, +0.19]
Head circumference (*z*-score)	T1	Continuous	364	-0.17 [-0.56, +0.22]
Head circumference (*z*-score)	T2	Discrete	380	-0.16 [-0.64, +0.31]
Head circumference (*z*-score)	T2	Continuous	380	-0.33 [-0.72, +0.05]
Intelligence (MPI)	T1	Discrete	285	-1.09 [-4.16, +1.98]
Intelligence (MPI)	T1	Continuous	284	+0.39 [-2.25, +3.04]
Intelligence (MPI)	T2	Discrete	348	-1.26 [-4.29, +1.77]
Intelligence (MPI)	T2	Continuous	348	-1.02 [-3.42, +1.38]

There is little consistency as to whether discrete or continuous measures have larger associations, and likewise for T1 versus T2 measures. In 8 comparisons of models with the same outcome and reporting time, 5 have a greater absolute value for the coefficient of the discrete measure than the continuous measure. When model *R*2 values are compared, continuous models have greater fit than the corresponding discrete model in 5 of 8 comparisons. For reporting time, T2 measures have the greater coefficient in 5 of 8 comparisons, whereas T1 measures have the greater *R*2 value in 6 of 8 comparisons.

## Discussion

We have found that among mothers in the Western Cape, as in other populations [4, 5, 6], mothers report drinking greater amounts prenatally when questioned after rather than during their pregnancy. By contrast, when answers are coded dichotomously as drinking or abstinent, mothers are more likely to say they drank when interviewed during pregnancy. Investigating associates of inconsistency, we see little association of having a new pregnancy with T2 drinking reports. Finally, we find only weak associations of all our drinking measures with child health outcomes, and the strengths of these associations do not systematically differ with respect to the scale (discrete or continuous) or the time (T1 or T2) of prenatal drinking measures.

### Change over time

Our findings point to the complexity of measuring prenatal drinking. Good measurement can depend on when mothers are asked as well as how mothers’ responses are coded. It is particularly surprising that dichotomous reports indicate a decrease over time whereas continuous reports indicate an increase. One possible explanation for this discrepancy is that women interviewed retrospectively tend to round small amounts to 0 and other amounts upwards, perhaps because of memory errors. Such an effect would be a sort of opposite of the finding in decision-making research that people overweight probabilities slightly more than 0 and underweight probabilities slightly less than 1 [24].

### Child outcomes

The weak associations of prenatal drinking reports with child outcomes are puzzling. One possible reason is that although our sample is large overall, we did not specifically recruit mothers who reported high drinking levels during pregnancy. Hence, among mothers who reported any drinking, we observed means of only 0.39 AA/day at T1 and 0.96 AA/day at T2, and only a few mothers reported heavy prenatal drinking (e.g., only 5 mothers at T1 and 18 mothers at T2 reported drinking at least 3 standard drinks or 1.8 AA/day). It follows that our findings do not say much about heavier prenatal drinkers, and it might not be realistic to expect strong effects on child health from the levels of prenatal drinking we observed.

The weak associations may indicate that these mothers’ reports about prenatal drinking are highly inaccurate. On the other hand, there could just as well be other, much larger sources of variation in body size and intelligence obscuring the true relationships. Ultimately, judging the accuracy of drinking reports requires some other measure for comparison, such as direct observation, informant report, or biomarkers, or at least a technique to assess dishonesty such as the bogus pipeline [25]. The lack of all these is a key limitation of this study. We found in a study of young men in nearby Cape Town [26] that only 61% of men who drank in the last three days, according to a urine test, admitted as much in an interview. Still less accurate self-report might be expected for the more dangerous and stigmatized activity of drinking while pregnant. Along with the inconsistency over time observed in this study, one could argue that self-report is simply not accurate enough for this population. Physiological measures may be necessary.

One might suspect that retrospective reports of prenatal drinking are determined mostly by current drinking [5]. That is, when asked after the fact about their prenatal drinking, mothers report their current drinking instead. However, we found in an American sample [14] that retrospective reports of prenatal drinking were more correlated with child head circumference (*r* = −.44) and number of physical anomalies (*r* = .39) than reports of current drinking were correlated with the same outcomes (*r*s = .21, −.14). Hence, retrospective reports do not seem to be reflections of current drinking habits alone.

### Limitations

A possible limitation of this study is that we measured drinking with a few short questions rather than a detailed interview or timeline follow-back procedure, which may have aggravated underreporting of drinking. Furthermore, there was wide variability in how long women had been pregnant when they were interviewed at T1 (the SD was 8.4 weeks), so T1 reports do not represent as uniform a group as could be desired. Lastly, while we considered continuous and dichotomous measures of drinking, we did not consider discrete measures with more than two levels, which could be on a nominal or ordinal scale. Perhaps these intermediate granularities could have advantages over fully continuous or dichotomous measures.

### Recommendations

Our best recommendation for future studies is that self-report should be conducted retrospectively and with a continuous measure. We say this because our findings for continuous measures are consistent with the American findings [4, 5, 6] that larger amounts are reported later, whereas dichotomous measures have shown less consistent patterns. We expect that because of stigma, reports of larger amounts will be more accurate, all other things being equal.

Continuous measures have a few additional advantages. First, it is straightforward to analyze a continuous measure dichotomously if a researcher wishes to, whereas if a variable was only measured dichotomously to begin with, there is no way to recover more granular information. Second, the possibility of dose-response effects (as suggested by our own findings of weak associations with child outcomes) and the additional difficulty that may be involved in convincing mothers to abstain from alcohol entirely rather than to cut back [11] suggest that the actual amounts pregnant women drink, as opposed to whether they drink at all, are worth our attention.

## Supporting information

S1 File The analytic data. (JSON)

S1 Table Sample sizes, *R*^2^ values, coefficients, and confidence intervals of the models for child outcomes. (CSV)
